# A retrospective cohort study of pregnancy outcomes during the pandemic period of the SARS‐CoV‐2 omicron variant: A single center's experience

**DOI:** 10.1002/ijgo.14312

**Published:** 2022-07-04

**Authors:** Ruairí Floyd, Samuel Hunter, Niamh Murphy, Stephen W. Lindow, Michael P. O'Connell

**Affiliations:** ^1^ Department of Obstetrics & Gynaecology Coombe Women and Infants University Hospital Dublin Ireland

**Keywords:** obstetric outcomes, omicron, pregnancy, SARS‐CoV‐2 infection

## Abstract

Our retrospective review revealed that antenatal infection with the Omicron variant is associated with minimal symptoms in vaccinated patients, minimal medical intervention, and good obstetric outcomes.

The SARS‐CoV‐2 Omicron B.1.1.529 variant became the dominant variant in Ireland by mid‐late December 2021.^1^ The WHO warned that Omicron was highly transmissible compared with SARS‐CoV‐2 Delta B.1.617.2, with an altered structure capable of vaccine‐mediated immune‐response evasion.^1^ Previously, Delta spread through the pregnant population with one in seven hospital admissions requiring intensive care, 98% of whom were unvaccinated.^2^ Maternal SARS‐CoV‐2 infection associations include doubled risk of stillbirth, increased incidence of small for gestational age, and a three‐fold increase in primarily iatrogenic preterm birth.^3^ The Royal College of Obstetricians & Gynecologists warned that Omicron may be associated with less severe disease than Delta, but more infectious, and still likely associated with adverse maternal and neonatal outcomes.^2^

We aimed to assess outcomes of antenatal infection with the Omicron variant between late December 2021 and March 2022. We retrospectively identified 44 women infected with SARS‐CoV‐2 on polymerase chain reaction (PCR) testing. Our laboratory does not report individual variants but over 90% of cases in the country at the time were the Omicron variant.^1^ The mean age of participants was 31 years (range, 18–41 years), of whom 48% were primiparous. Of the cohort, 48% were unvaccinated, with 2%, 39%, and 11% having received 1, 2, or 3 vaccines, respectively. All infections were PCR‐confirmed with 2% of patients reinfected within the same pregnancy. Significant medical history was noted in 66% of participants, with significant current comorbidity in 39%. Mean body mass index was 26.4 kg/m^2^ (range, 20.2–42.2 kg/m^2^).

A total of 32% of patients had documented symptoms (Table 1). In symptomatic patients, the duration of symptoms ranged from 2 to 9 days, although this was largely unrecorded in outpatients. No patients required supplemental oxygen, ventilation, steroids, or antiviral agents. A total of 9% of patients required hospital admission with no high‐dependency or intensive care unit admissions. Reasons for admission included management of hyperemesis and pyrexia. The mean duration of admission was 3.75 days.

**TABLE 1:** SARS‐CoV‐2 symptoms based on vaccination status

Symptoms	Total (*N* = 44)	Fully vaccinated (*n* = 5)	Partially vaccinated^a^ (*n* = 18)	Unvaccinated (*n* = 21)
Cough	6 (14)	0 (0)	3 (7)	3 (7)
Pyrexia	3 (7)	0 (0)	1 (2)	2 (5)
Sinusitis	3 (7)	0 (0)	2 (5)	1 (2)
Fatigue	3 (7)	1 (2)	2 (5)	0 (0)
Dyspnea	3 (7)	0 (0)	1 (2)	2 (5)
Nausea and/or vomiting	2 (5)	0 (0)	0 (0)	2 (5)
Significant ketonuria	2 (5)	0 (0)	0 (0)	2 (5)
Headache	1 (2)	0 (0)	1 (2)	0 (0)
Sore throat	1 (2)	0 (0)	0 (0)	1 (2)
Reduced fetal movements	1 (2)	0 (0)	1 (2)	0 (0)
Abdominal pain	0 (0)	0 (0)	0 (0)	0 (0)
Diarrhea	0 (0)	0 (0)	0 (0)	0 (0)
Hypogeusia	0 (0)	0 (0)	0 (0)	0 (0)
Anosmia	0 (0)	0 (0)	0 (0)	0 (0)
“Mild symptoms”	4 (9)	2 (5)	1 (2)	1 (2)
Requiring admission	4 (9)	0 (0)	2 (5)	2 (5)

*Note*: Values are expressed as number (percentage).

^a^ Partially‐vaccinated patients had received 1 or 2 doses without having received a booster vaccination.

There were no preterm deliveries or stillbirths. Delivery was expedited in 86% of cases. Spontaneous vaginal delivery was recorded in 64%, ventouse delivery in 9%, emergency cesarean section in 16%, and elective cesarean section in 11%. Mean birth weight was 3403 g (range, 2775–4345 g).

Our results show that the Omicron variant in pregnancy is associated with mild symptoms and minimal requirement for medical intervention. The aim of this study is to add to the minimal data available on pregnancy outcomes during the Omicron variant pandemic period. Our data are similar to the only report to date reporting minimal symptoms in vaccinated patients and low burden of symptoms in unvaccinated patients.^4^ Our data are limited because of the absence of specific testing for the Omicron variant. Currently, we are assessing the effects of Omicron in a larger sample with fetal outcomes to fully understand Omicron's effect on pregnancy.

## AUTHOR CONTRIBUTIONS

Ruairí Floyd, Samuel Hunter, Niamh Murphy, Stephen Lindow, and Michael O'Connell planned the study. Stephen Lindow and Michael O'Connell designed the data collection sheet. Ruairí Floyd and Samuel Hunter were involved in chart review and data collection. Ruairí Floyd conducted the review of the literature and wrote the paper. Samuel Hunter, Niamh Murphy, Stephen Lindow, and Michael O'Connell reviewed and edited the paper. Ruairí Floyd submitted the review.

## CONFLICT OF INTEREST

The authors have no conflicts of interest.

## Data Availability

The data that support the findings of this study are available from the corresponding author on reasonable request.

## References

[1] World Health Organization . Classification of Omicron (B.1.1.529): SARS‐CoV‐2 variant of concern. https://www.who.int/news/item/26‐11‐2021‐classification‐of‐omicron‐(b.1.1.529)‐sars‐cov‐2‐variant‐of‐concern. Accessed June 24, 2022.

[2] Royal College of Obstetricians & Gynaecologists . Coronavirus (COVID‐19) infection in pregnancy: Information for healthcare professionals. Version 14.3. Published January 11, 2022.

[3] Allotey J , Stallings E , Bonet M , et al. Clinical manifestations, risk factors, and maternal and perinatal outcomes of coronavirus disease 2019 in pregnancy: living systematic review and meta‐analysis. BMJ. 2020;370:m3320. 32873575 10.1136/bmj.m3320 PMC7459193

[4] Ilter PB , Prasad S , Berkkan M , et al. Clinical severity of SARS‐CoV‐2 infection among vaccinated and unvaccinated pregnancies during the omicron wave. Ultrasound Obstet Gynecol. 2022;59:560‐562. 35229932 10.1002/uog.24893 PMC9111183

